# Same-strand overlapping genes in bacteria: compositional determinants of phase bias

**DOI:** 10.1186/1745-6150-3-36

**Published:** 2008-08-21

**Authors:** Niv Sabath, Dan Graur, Giddy Landan

**Affiliations:** 1 Department of Biology and Biochemistry, University of Houston, Houston, TX 77204, USA

## Abstract

**Background:**

Same-strand overlapping genes may occur in frameshifts of one (phase 1) or two nucleotides (phase 2). In previous studies of bacterial genomes, long phase-1 overlaps were found to be more numerous than long phase-2 overlaps. This bias was explained by either genomic location or an unspecified selection advantage. Models that focused on the ability of the two genes to evolve independently did not predict this phase bias. Here, we propose that a purely compositional model explains the phase bias in a more parsimonious manner. Same-strand overlapping genes may arise through either a mutation at the termination codon of the upstream gene or a mutation at the initiation codon of the downstream gene. We hypothesized that given these two scenarios, the frequencies of initiation and termination codons in the two phases may determine the number for overlapping genes.

**Results:**

We examined the frequencies of initiation- and termination-codons in the two phases, and found that termination codons do not significantly differ between the two phases, whereas initiation codons are more abundant in phase 1. We found that the primary factors explaining the phase inequality are the frequencies of amino acids whose codons may combine to form start codons in the two phases. We show that the frequencies of start codons in each of the two phases, and, hence, the potential for the creation of overlapping genes, are determined by a universal amino-acid frequency and species-specific codon usage, leading to a correlation between long phase-1 overlaps and genomic GC content.

**Conclusion:**

Our model explains the phase bias in same-strand overlapping genes by compositional factors without invoking selection. Therefore, it can be used as a null model of neutral evolution to test selection hypotheses concerning the evolution of overlapping genes.

**Reviewers:**

This article was reviewed by Bill Martin, Itai Yanai, and Mikhail Gelfand.

## Background

**Figure 1:**
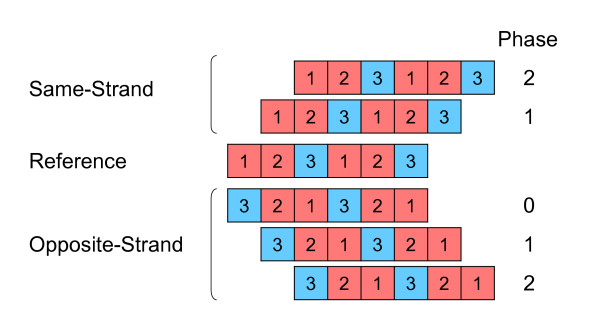
Orientations and phases of gene overlap. Genes can overlap on the same strand and on the opposite strand. The reference gene in a pair of overlapping genes is called phase 0. Same-strand overlaps can be in two phases (1 and 2); opposite-strand overlaps can be in three phases (0, 1, and 2). First and second codon positions, in which ~5% and 0% of the changes are synonymous, are marked in red. Third codon positions, in which ~70% of the changes are synonymous, are marked in blue.

Overlapping genes were found in all cellular domains of life, as well as in viruses [1-3]. Overlapping genes are thought to have unique evolutionary constraints [4,5] and regulatory properties [6,7]. Genes can overlap on the same strand (→ →) or on the complementary strand ("tail-to-tail" → ←, or "head-to-head" ← →, Figure 1). Different nomenclatures have been used in the literature to denote "same-strand" ("unidirectional," "codirected," "parallel," and "tandem"), "tail-to-tail" ("convergent," "anti-parallel," and "end-on"), and "head-to-head" ("divergent" and "head-on") overlapping genes [8-11]. Here, we use the self-explanatory terms "same-strand" and "opposite-strand" overlapping genes.

In bacteria, overlaps on the same strand are by far the most abundant [10,11], most likely because, on average, 70% of the genes in bacterial genomes, are located on one strand [9]. Same-strand overlaps occur in frameshifts of one nucleotide (phase 1) or two nucleotides (phase 2). Overlaps in the same frame (phase 0) are rare [11], and since the reading frame is unaffected, they may be thought of as genes with alternative initiation or termination sites rather than overlapping genes. Phase-0 overlaps are not dealt with here. Several studies have shown that there are significant differences between the frequencies of phase-1 and phase-2 overlapping genes [3,8,11]. Overlapping-gene pairs, in which the overlap sequence is of length one to five bases (short overlaps), are abundant in phase 2, but rare in phase 1. This difference is dictated by the sequence of termination codons of the upstream gene [8]. Since none of the stop codons (TGA, TAG, and TAA) ends in AT, GT, or TT (needed to create the initiation codons ATG, GTG or TTG in phase-1 two-nucleotide overlap) or start with G (needed to create an initiation codon in phase-1 five-nucleotide overlap), short phase-1 overlaps can only use alternative initiation codons. In contrast, as far as long overlaps (seven nucleotides or longer) are concerned, phase-1 overlapping gene pairs are more frequent than those of phase 2 [8,11]. Cock and Whitworth [8] suggested that the phase bias in long overlaps is due to some unspecified selective advantage of phase-1 over phase-2 overlapping genes. They also hypothesized that since the bias was found to be universal and independent of gene function, it might be a property of gene location. Krakauer [4] introduced a model in which the frequencies of overlapping genes in different phases are determined by their level of interdependence with respect to selective constraints. That model assumes an adaptive advantage for overlapping genes in evolvable phases [4]. For example, in phase-1 opposite-strand overlaps, in which the second codon position of one gene corresponds to the third codon position of the second gene (and vice versa), the freedom of each gene to evolve independently is maximized [4] (Figure 1). Indeed, Rogozin et al. [12] found that among opposite-strand overlaps in bacteria, the least constrained overlap phase (phase 1) was the most abundant. Kingsford et al. [13] explained this phase distribution in opposite-strand overlapping genes by the frequency of reverse-complementary stop codons in coding sequences. For same-strand overlaps, phase-1 and phase-2 overlaps have equal selective constraints and are predicted by this model, to occur in equal frequencies [4].

Previous studies [9,11] have found that the number of overlapping genes in bacterial genomes is positively correlated with the number of genes, implying that gene overlap may be mainly the result of accidental or random "trespassing" of one gene into another. There can be two scenarios for the creation of same-strand overlapping genes from pre-existing neighboring genes: (1) a mutation in the termination codon of the upstream gene, resulting in an extension of the gene downstream to the first in-frame termination codon and (2) a mutation in the initiation codon of the downstream gene, resulting in an extension of the gene upstream to the first in-frame functional initiation codon [9]. As in point mutations, where the effect of nonsynonymous mutation is expected to be stronger than that of synonymous ones, the impact of mutations that cause extension is expected to vary according to the length of the extension. Since most mutations are deleterious, long extensions of genes are expected to be under stronger purifying selection than short ones [13] and the frequency of initiation and termination codons in a certain phase is an upper-limit constraint to the possible number of overlapping genes in that phase.

Here, we tested the influence of initiation- and termination-codon frequencies as well as genomic GC-content on the number of overlapping genes in the two phases.

## Methods

**Figure 2:**
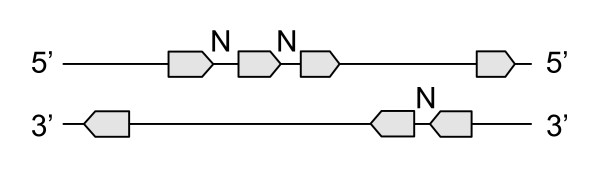
Same-strand neighboring gene pairs (marked with the letter N) are defined as two adjacent genes that are located on the same strand and in between them there are no genes on the opposite strand.

Data of overlapping genes from 167 bacterial genomes that employ the universal genetic code were acquired from the BPhyOG overlapping-genes database [14]. Same-strand overlapping genes in each genome were classified according to phase and the length of the intersecting segment. We defined overlap frequency as the number of same-strand overlapping genes divided by the number of same-strand neighboring gene pairs (i.e., adjacent genes, which are located on the same strand and in between them there are no genes on the opposite strand, Figure 2) in the genome. In our analysis, we explicitly ignored recombination and therefore we used the number of same-strand neighboring gene pairs, rather than the number of genes, because a neighboring gene pair located on opposite strands cannot become overlapping on the same strand as a result of point mutation. Short overlaps (two and five bases in phase 1 and one and four bases in phase 2) were dealt separately from long overlaps of seven bases or longer.

The coding sequences of the studied genomes were downloaded from NCBI. Codon and amino-acid frequencies, as well as initiation and termination codon frequencies in phase 1 and phase 2, were calculated from the coding sequences of each genome. We denote the frequency of a codon or a group of codons with a superscript for the codon's phase and a subscript for the codon. For example, $f_{ATG}^{1}$ denotes the frequency of ATG in phase 1 and $f_{NAT}^{0}$ denotes the frequencies of codons in phase 0 that end in AT, where N denotes any of the four nucleotides. The expected frequencies of each start and stop codons are calculated as the products of the frequencies of the codons that combine them, i.e., $f_{NAT}^{0}\times f_{GNN}^{0}$ and $f_{NNA}^{0}\times f_{TGN}^{0}$ for ATG in phase 1 and phase 2, respectively. If the codons frequencies in phase 1 and phase 2 are primarily determined by the frequencies of the codons in phase 0 that combine them, the expected frequencies would match the observed frequencies.

## Results

We identified 71,210 same-strand overlapping gene pairs (Table 1). Short overlaps (of length two or five bases) are rare in phase 1. In our sample, we found only 18 phase-1 short overlaps (0.08%, Table 1). In contrast, the majority of phase-2 overlaps are of length one or four bases (20% and 65%, respectively).

**Table 1:** Number of same-strand overlapping genes.

	Short overlaps (1–5 bases)	Long overlaps (7 bases or more)	Total
Phase 1	18	21,550	21,568
Phase 2	42,177	7,465	49,642
Total	42,195	29,015	71,210

We used 167 bacterial genomes from Luo et al. [14]. Nine genomes GenBank:NC_000908, GenBank:NC_000912, GenBank:NC_002162, GenBank:NC_002771, GenBank:NC_004432, GenBank:NC_004829, GenBank:NC_005364, GenBank:NC_006055, and GenBank:NC_006908 that do not employ the universal genetic code were excluded.

**Figure 3:**
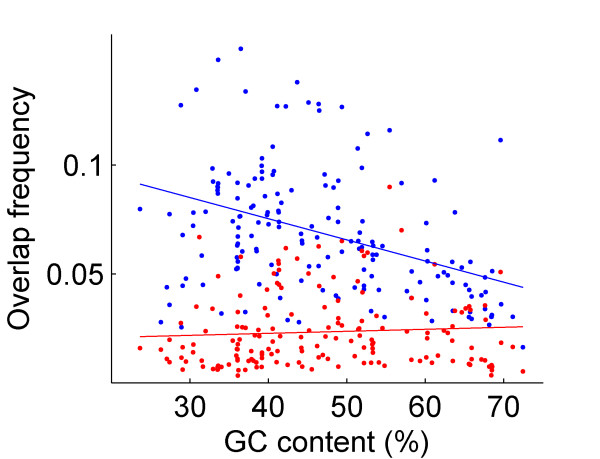
Frequency of overlapping genes in 167 bacterial genomes plotted against genomic GC content. Long phase-1 overlaps are marked in blue. Long phase-2 overlaps are marked in red.

**Figure 4:**
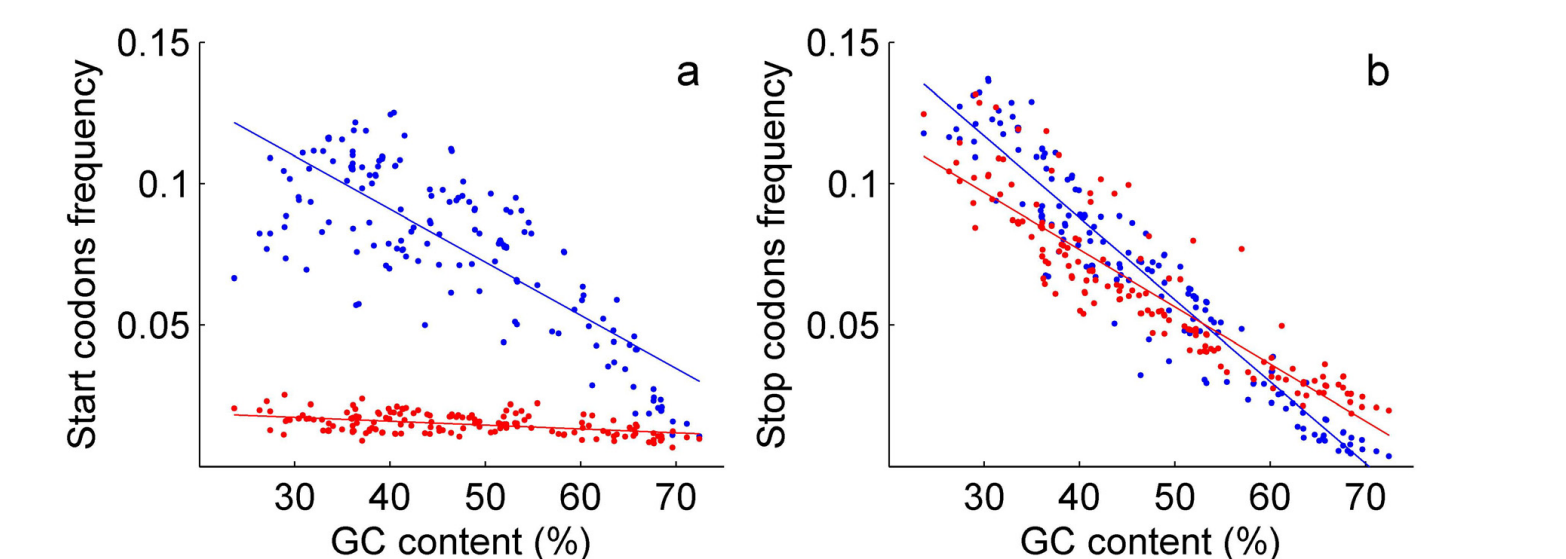
a. Start codon frequencies in phase-1 (blue) and phase-2 (red) reading frames plotted against genomic GC content. b. Stop codon frequencies in phase-1 (blue) and phase-2 (red) reading frames plotted against genomic GC content.

The frequency of long phase-1 overlaps exceeds that of long phase-2 overlaps by a factor of almost 3 (Table 1, Figure 3, two-sample paired Student t-test, p < 0.001). The frequency of long phase-1 overlaps is negatively correlated with genomic GC content (Figure 3, *r *= -0.39, *p *< 0.001). In contrast, the correlation between the frequency of long phase-2 overlaps and GC content is not significant (*p *= 0.4). The frequencies of start and stop codons in phase 1 and phase 2 in the coding regions of the genomes are presented in Figure 4. Pooling together phase 1 and phase 2, the frequency of stop codons (average of 13.16%) is significantly higher than that of start codons (average of 9.36%, two-sample paired Student t-test, *p *< 0.001). We found that the frequency of start codons in phase 1 is significantly higher than that in phase 2 by a factor of 5.2 on average (Figure 4a, two-sample paired Student t-test, *p *< 0.001). There is no significant difference between the frequencies of stop codons in the two phases (Figure 4b, two-sample paired Student t-test, *p *= 0.13). These results suggest that the difference between the number of long overlaps in phase 1 and phase 2 is primarily influenced by the frequencies of start codons in the two reading frames.

**Figure 5:**
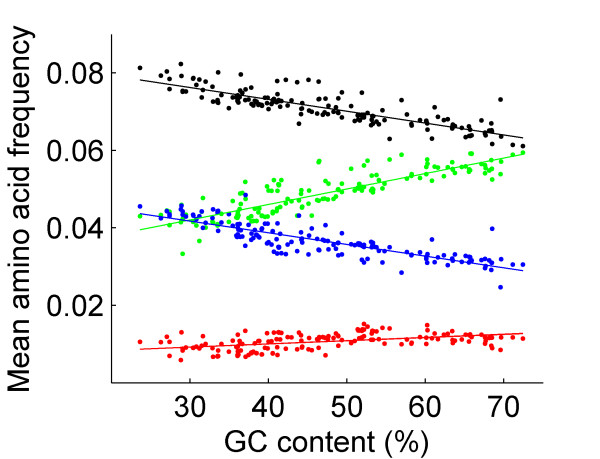
Mean frequencies of groups of amino acids in the 167 bacterial genomes plotted against genomic GC content. Mean frequency of amino acids, which are encoded by TGN, NAT, NGT, or NTT codons, are marked in red, blue, green, and black, respectively. NAT, NGT, and NTT codons may lend a dinucleotide to one of the start codons in phase 1. TGN codons may lend a dinucleotide to one of the start codons in phase 2.

The difference in start codon frequencies between phase 1 and phase 2 can be explained by the codons in phase 0 that may potentially lend a dinucleotide to a start codon (ATG, GTG, and TTG) in each of the phases. In phase 2, all start codons consist of phase-0 TGN codons, which may lend TG to form a phase-2 start codon. One of these codons, TGA, is a stop codon that cannot be a part of long overlap. The remaining three codons (TGT, TGC, TGG) encode for two amino acids (cysteine and tryptophan), which are among the rarest in protein-coding genes, with a mean frequency of ~1% (Table 2). In contrast, in phase 1, the amino acids coded by NAT, NGT, and NTT codons that may lend a dinucleotide to one of the start codons (ATG, GTG, and TTG, respectively), are found in moderate to high frequencies in proteins (Table 2). Interestingly, the abundance of NAT-, NGT-, and NTT-encoded amino acids is inversely correlated with the frequency of start codons (Table 2). Moreover, amino acids encoded by NAT codons which can form the most common start codon, ATG, appear in lower frequencies than amino acids encoded by NGT- and NTT-encoded amino acids. For all bacteria and for all GC contents the frequencies of amino acids coded by TGN codons are lower than each of the amino acid groups encoded by NAT, NGT, and NTT (Figure 5, all pairwise two-sample paired Student t-tests, *p *< 0.001).

**Table 2:** Codons in phase 0 that may lend a dinucleotide to form a start codon in phase 1 and phase 2. The usage of each start codon in (a) all genes; (b) the downstream gene of long phase-1 overlaps; and (c) the downstream gene of long phase-2 overlaps, is noted.

Start Codon (usage in: all genes, phase1, phase 2)	Phase	Codon Group	Amino Acids	Mean amino acid frequency
ATG (^a^77%, ^b^73%, ^c^64%)	1	NAT	Tyr, His, Asn, Asp	3.67%
	2	TGN	Cys, Trp	1.06%
GTG (^a^14%, ^b^15%, ^c^23%)	1	NGT	Cys, Arg, Ser, Gly	4.87%
	2	TGN	Cys, Trp	1.06%
TTG (^a^9%, ^b^12%, ^c^14%)	1	NTT	Phe, Leu, Ile, Val	7.12%
	2	TGN	Cys, Trp	1.06%

Thus, consideration of the number of amino acids and their frequencies alone will lead us to expect start codons to occur much more frequently in phase 1 than in phase 2. However, the difference in amino acids usage does not provide a very good fit to the observed frequencies. This can be achieved by a more detailed compositional argument, one that is based on codon frequencies. Such a model will accommodate differences in GC content and codon usage among the bacteria under study. We found that the frequencies of the codons that combine to form start and stop codons (e.g., $f_{NAT}^{0}\times f_{GNN}^{0}$ and $f_{NNA}^{0}\times f_{TGN}^{0}$ for ATG), are strongly correlated with the frequencies of start and stop codons in both phases, as well as with genomic GC content (Table 3).

**Table 3:** The correlation between the frequency of frame-shift start and stop codons and (a) their expected frequencies; and (b) the genomic GC content. All correlations are significant at the *p *< 0.001 level (sample size is 167).

Frame-Shift Codon		Phase	Combining Codons	^a^Correlation Observed-Expected	^b^Correlation Observed-GC%
Start	ATG	1	N**AT,G**NN	0.96	-0.84
		2	NN**A,TG**N	0.89	-0.76
	GTG	1	N**GT,G**NN	0.94	-0.34
		2	NN**G,TG**N	0.86	0.80
	TTG	1	N**TT,G**NN	0.96	-0.80
		2	NN**T,TG**N	0.87	-0.70

Stop	TAA	1	N**TA,A**NN	0.98	-0.87
		2	NN**T,AA**N	0.97	-0.93
	TAG	1	N**TA,G**NN	0.96	-0.89
		2	NN**T,AG**N	0.90	-0.84
	TGA	1	N**TG,A**NN	0.86	0.51
		2	NN**T,GA**N	0.92	-0.84

To control for potential annotation errors, we used a subset of overlapping genes that were not annotated as "hypothetical," "putative" or "pseudogene" in the NCBI genome data. This subset of overlapping genes, which we assume to be more accurately annotated, contains 31,767 gene pairs (45% of the complete set). As in the complete set, the frequency of long phase-1 overlaps exceeds the frequency of long phase-2 overlaps by a factor of 3.1 and the frequency of long phase-1 overlaps is negatively correlated with genomic GC content (*r *= -0.28, *p *< 0.001), whereas the frequency of long phase-2 overlaps is not (*p *= 0.6). Therefore, the influence of misannotation seems not to be significant.

## Discussion

Understanding the distribution of overlapping genes in different phases is a key step towards distinguishing between the effects of selection and mutation on the evolution of overlapping genes. Krakauer [4] showed that overlapping genes in different orientations and phases differ in the freedom for each gene to evolve independently. Therefore, he suggested that the variation in selective constraints would be reflected in the frequency of the overlap phases. In the case of same-strand overlapping genes, his model predicted no difference between the frequency of phase-1 and phase-2 overlaps [4]. However, in agreement with previous studies [3,8,11], our results indicate a preponderance of long phase-1 overlaps over long phase-2 overlaps. Cock and Whitworth [8] attributed the difference between the number of long overlaps in the two phases to either gene location or to an unspecified selective advantage. These hypotheses cannot be quantifiably tested.

Considering the two scenarios for the creation of same-strand overlapping genes, we showed that the phase bias in long overlaps might be attributed to a great extant to overlaps created by 5'-end mutation of the downstream gene. Since there is purifying selection against long overlaps, the frequency of start codons in phase 2 constrains the number of overlap that can be created in that phase and leads to the phase bias. In addition, we showed that the difference in start codon frequencies between phase 1 and phase 2 is dictated by the frequencies of amino acids whose codons may combine to form start codons in the two phases. Finally, the dependency of frame-shift start and stop codons on species-specific codon usage result in a correlation between long phase-1 overlap frequency and genomic GC content.

Although our model explains the phase bias in overlap frequency, we do not have a full explanation for the absence of correlation between GC content and long phase-2 overlaps as expected from the frequency of frame-shift start and stop codons. This correlation is expected to have lower statistical significance than that of phase-1 overlaps because of the smaller sample size, but it is also possible that other factors affect the potential for overlap as well. A more complex compositional model for overlapping genes frequency, might include the length distribution of overlaps, the frequencies of regulatory elements (e.g., Shine-Delgarno sequences) and the strand-specific composition bias, since bacterial genomes have an asymmetrical chirochoric base composition [15-17].

The wide abundance of overlapping genes and the straightforward definition of phase evolvability make the phase distribution of overlapping genes an interesting case study. If evolvability is selected for, the expectation is for a positive correlation to exist between the frequency of an overlap phase and its evolvability. Evolvability considerations predict phase-1 and phase-2 overlaps to occur at equal frequencies [4]. Therefore, our data does not support a role for evolvability in the evolution of same-strand overlapping genes.

Fukuda et al. [9] examined homologous overlapping genes in related bacterial species and found that the rate of accumulation and degradation of overlapping pairs is higher for overlaps caused by mutation at the 3'-end of the upstream gene compared to overlaps caused by mutation at the 5'-end of the downstream gene. The difference in rates was suggested to be a result of an evolutionary constraint imposed on the 5'-end of genes [9]. Our model predicts a difference in these rates simply because of the higher frequency of frame-shift stop codons compared to the frequency of frame-shift start codons. It would be interesting to test whether the rate difference of accumulation and degradation of overlapping gene pairs in the two scenarios holds even when accounting for the difference in frequency of frame-shift stop codons compared to frame-shift start codons.

The high frequency of frame-shift stop codons was previously suggested to be under positive selection for minimization of frame-shift translation errors [18,19]. We found that the frequency of frame-shift stop codons is strongly correlated with genomic GC content leading to AT-rich genomes having five times more frame-shift stop codons than GC-rich genomes. Therefore, it seems that the mutation pattern is a major player in determining frame-shift stop-codon frequencies, while selection does not seem to play a major role.

Viral genomes also exhibit high frequencies of overlapping genes. In a study of RNA viruses, Belshaw et al. [20] distinguished between internal overlaps, in which one gene is embedded within the other, and terminal overlaps. For internal overlaps, it was found that, similar to bacteria, there is a predominance of phase-1 overlaps [20]. In the case of terminal overlaps, Belshaw et al. [20] reported no frequency difference between phase 1 and phase 2. However, Belshaw et al. [20] did not distinguish between short overlaps, in which phase-1 overlaps are extremely rare, and long overlaps. We showed that at least as far as bacteria are concerned, pooling short and long overlaps together results in obscuring the pattern for long overlaps (Table 1). Therefore, the similar frequencies of overall overlaps in phase 1 and phase 2 in RNA viruses [20], suggests that the phase bias in long overlaps was most likely unnoticed.

## Conclusion

1. The phase-distribution of same-strand overlapping genes in bacteria is determined by the frame-shift frequencies of start and stop codons in protein-coding genes.

2. The predominance of long phase-1 overlaps results from a lower frequency of start codons in phase 2 that limits the potential overlaps created by an upstream extension of the downstream gene.

3. The difference in the frequency of start codons is dictated by the abundance of those amino acids that are encoded by codons that combine to form start codons in phase 1 and phase 2. This difference is conserved among all the bacterial genomes in the study.

4. The variability of codon usage across bacterial genomes leads to a correlation between long phase-1 overlaps and genomic GC content.

5. Our model explains the phase bias in same-strand overlapping genes by compositional factors without invoking selection. Therefore, it can be used as a null model of neutral evolution for testing selection hypotheses affecting the evolution of overlapping genes.

## Competing interests

The authors declare that they have no competing interests.

## Authors' contributions

NS performed the analyses and wrote the draft manuscript. DG and GL contributed to the interpretation of the results and the final version.

## Reviewers' comments

### Reviewer's report 1

Review by Bill Martin, University of Dusseldorf.

This is an interesting and straightforward paper showing that the main patterns shown by overlapping genes can be simply explained with constraints posed by base compositional factors and the nature of the genetic code. I had not thought much about overlapping genes and the conundrum that they entail, and I suspect that many other readers have not either, so the present paper was a very worthwhile read for me and I suspect that others will see it similarly.

### Reviewer's report 2

Review by Itai Yanai, Department of Biology, Technion – Israel Institute of Technology

1) In this paper, Sabath et al. propose a convincingly simple explanation for a known genomic bias without recourse to positive selection. This is a significant achievement and a sobering one too given that it offers a minimal mechanism to a process where only complicated explanations were previously available. The coding regions of neighboring same-strand genes sometimes overlap, and for this overlap to consist of a different open reading frame a frame-shift of one (phase 1) or two (phase 2) base-pairs may be introduced. While it might be expected that both phases occur equally frequently, Sabath et al. confirm, using a large set of 167 genomes, the previously reported observation that long overlaps (=7 bp), phase 1's are favored 3 to 1 to phase 2's. This trend has been previously attributed to an unknown selective advantage or genomic location; however the authors here provide evidence for the preference of phase 1 codons from a simple base-pair compositional perspective.

2) The results can be essentially seen here as two themes: 1. Sabath et al. show that when examining coding regions, the codons in phase 1 contain more start codons than in the phase 2; and 2. that this trend holds across 167 genomes, although an impressive dependency with GC content is also revealed. For the former, the authors make the argument that the formation of a start codon in phase 2 is less probably since it requires rare phase 0 codons. This is a simple and brilliant explanation that appears well supported by the data. It is an explanation which does not require special selective biases and I fully support the authors claim that this is a neutral model which ought to be considered the null-hypothesis for the formation of overlapping genes.

3) As noted by the authors however, there seems to be another layer to this puzzle that remains unsolved. Throughout, Sabath et al. demonstrate the correlations across an axis of GC content, where genomes with a high GC content contains less fraction overlapping genes, of start codons in phase 1, and of stop codons in both phase 1, and 2. These strong correlation are the elephant in the room, especially contrasted with the lack of correlation of GC content with phase-2 long overlaps. It would be interesting to test whether frequent phase 0 codons lead to more popular codons in phase 1 than in phase 2. Since a gene with less frequent codons may also have low expression, purifying selection would tend to select against overlaps with unpopular codons. This analysis would generalize Sabath et al.'s analysis of the start/stop codons to the entire genetic code.

#### Author's response

*The lack of correlation between phase-2 long overlaps and genomic GC content is, indeed, unresolved. When trying to resolve this issue, one has to keep in mind that the observed negative correlation between start and stop codons and GC content is a result of these codons being AT rich in sequence. However, this overall negative correlation contains particular positive correlations between GC content and some phase-0 codons that combine to yield start or stop codons in phase 1 and phase 2. For example, there is a negative correlation between GC content and the start codons ATG, GTG, and TTG in phase 2, whereas, the correlation between GC content and phase-0 TGC and TGG codons that may combine to yield a start codon in phase 2 is positive (data not shown). Unfortunately, this issue cannot be simply resolved by focusing on overlaps with one start codon at a time, since the factors governing start codon usage are not well understood for either overlapping or non-overlapping genes. The suggestion that the frequencies of overlap phases are influenced by codon-bias in phase-0 codons is important and should be studied in the future. In fact, as noted in the discussion, it would be important to consider other compositional factors (such as the length distribution of overlaps, the frequencies of regulatory elements, and the strand-specific composition bias) as well*.

4) On a final note, I do not agree with the authors statements on the evolvability of overlap in the Discussion section. Sabath et al. write: "If evolvability is selected for, the expectation is for a positive correlation to exist between the frequency of an overlap phase and its evolvability. Evolvability considerations predict phase-1 and phase-2 overlaps to occur at equal frequencies [4]. Therefore, our data does not support a role for evolvability in the evolution of same-strand overlapping genes." It is not clear what exactly is meant by evolvability in this context, and why an equal frequency among the phases would support this. I would have expected the authors to conclude here that evolvability is an inappropriate issue when discussing overlapping genes since the evidence provided here point to a predominantly neutral process.

#### Author's response

*For better or worse, the topic of evolvability of biological entities has been a subject of great interest in the recent years [reviewed in *[21]]. *However, the quantification of evolvability has been a difficult task. Overlapping genes are unique in that their evolvability can be quantified objectively. A biological system is evolvable if it can acquire novel functions through genetic change. In the case of overlapping genes, evolvability was defined as the degree in which each of the overlapping genes can evolve independently *[4], *i.e., the proportion of changes that are nonsynonymous in one gene and synonymous in the overlapping gene. Given that the three codon-positions are different in the proportion of changes that are synonymous (~5%, 0%, and ~70% for first, second, and third codon positions, respectively), phase evolvability depends on the combination of the three codon-positions in the two overlapping genes. For example, opposite-strand phase 2 is the least evolvable phase with the third codon position in one gene corresponds to the third codon position of the second gene (and vice versa), leading to maximization of the proportion of changes that are nonsynonymous in both genes *(Figure 1). *In contrast, opposite-strand phase 1 is the most evolvable phase, with the second codon position of one gene corresponds to the third codon position of the second gene (and vice versa) which maximizes the proportion of changes that are nonsynonymous in one gene and synonymous in the overlapping genes. Krakauer *[4]*suggested that overlapping genes in evolvable phases have an adaptive advantage over overlapping genes in less evolvable phases, since they allow for higher degrees of independent evolution. Therefore, he predicted a positive correlation between the frequency of an overlap phase and its evolvability *[4]. *Indeed, Rogozin et al*. [12]*found that among opposite-strand overlaps in bacteria, the least constrained overlap phase (phase 1) was the most abundant. This result was later questioned by Kingsford et al*. [13]*who used a similar approach to ours. In the case of same-strand overlaps, phase-1 and phase-2 overlaps have equal degree of evolvability *[4]. *The reason is that from the point of view of one gene, there is an equivalence of overlap phase. For example, if gene A overlaps gene B on the same strand in phase 1, than gene B overlaps gene A in phase 2. Therefore, phase-1 and phase-2 overlaps are predicted by this model to occur at equal frequencies. Since our data shows unequal frequencies of phase 1 and phase 2, evolvability does not seem to play an important role in the evolution of same-strand overlapping genes*.

### Reviewer's report 3

Review by Mikhail Gelfand, Department of Bioinformatics, Institute of Information Transfer Problems

1) It is common knowledge that in many cases it is much more difficult to prove a negative result than a positive one. Thus, the authors have set themselves a hard problem: to show that the frequencies of gene pairs overlapping in different frames can be explained by simple consideration of amino acid frequencies and codon usage and do not require more complicated evolutionary explanation.

#### Author's response

*Any scientific explanation should make as few assumptions as possible. We provided an explanation for the phase bias in same-strand overlapping genes that is simpler than previous ones and does not invoke selection for phase of overlap. A more complicated model will only be required if it can explain significantly more of the variation in the observed data than our simple model. In this case, the more complex model (i.e., that overlap phase frequency is determined by selective constraints) fails to explain the data and can, therefore, be discarded*.

2) While the point is well taken and the approach clearly interesting, there still seem to be some technical issues that have not been addressed. Of course, the main problem plaguing all large-scale genome analysis projects is reliance on existing annotations: one may find himself studying idiosyncrasies of annotation software rather than biologically relevant features. For some analyses the authors exclude genes annotated as hypothetical, but this does not guarantee that gene starts have been predicted correctly.

#### Author's response

*Annotation errors are a major concern in any computational analysis. Our approach of using a subset of genes for which there is higher confidence in the annotation is common in the literature in general, as well as in studies that deal with overlapping genes (e.g*., [11].).

3) At that, it is noteworthy that all non-trivial observations have been made for 5'-extensions, but not 3'-extension: it is fairly easy to mispredict the start codon (some annotation projects routinely consider the most distal codon to serve as the start), but not the stop codon.

I do not see an easy way out of this difficulty. One possible control is to consider separately overlaps caused by 5'-most start codons for the downstream gene (open reading frame) and internal start codons.

#### Author's response

*Our observations on 5'-extensions are not based on the annotation of start codons, but on the observed frequency of start codons in phase 1 and phase 2 of coding sequences. This difference in the frequency of start codons explains the difference in the frequency of long same-strand overlapping genes*.

4) Another approach is much more time-consuming, but it might provide interesting biological insight per se. The authors state that overlaps are caused by mutations in either start or stop codons. For the stop codons this should be not very difficult to trace to these mutations to specific branches of the evolutionary tree. Then the entire analysis might be repeated for the overlaps where the causing mutation is known. It is likely that it would seriously decrease the sample size, but it would also make the sample much more reliable. In particular, one might consider separately established overlaps persisting for some time and very recent overlaps caused by species-specific mutations (or, for that matter, sequencing errors).

With start codons it might be more difficult. Indeed, one has to consider separately two types of mutations. One is the loss of a pre-existing start codon, and this can be treated in a manner similar to the one when stops are considered. A useful addition would be considering separately cases where there are candidate start codons upstream (in the previous reading frame, either on the same strand, or the complementary strand) and when candidate start codons can be found within the gene whose original start codon is mutated. The second type of mutations is gain of function, that is, emergence of a new functional upstream start codon. However, in this case it would be very difficult to prove by purely computational means that the new start really functions.

There are also other possibilities for a more detailed analysis. A common problem for all of them is that they require considerable effort to prove a rather simple point, and thus it is not clear whether they are worth pursuing.

#### Author's response

*We agree that a phylogenetic approach may be beneficial. Unfortunately, the phylogenetic topology of bacteria is unresolved, so that a phylogenetic approach may introduce a new source of error into the analyses*.

5) Another important problem is, however, necessary to be addressed, as it clearly lies in the framework of the suggested approach. The point is, for a new upstream codon to be functional, it needs to occur in the same open reading frame as the old one, that is, there should be no stop codons in the region between the new and old starts. Since the frequency of candidate stop codons is not the same in the two shifted reading phases of the upstream gene, this might influence the general conclusions made in the paper. It looks like the authors have something like that in mind when they write about "stronger purifying selection" in long extensions, but this point is never quantified, and the applied term looks somewhat misleading and inviting further criticisms: if there is stronger purifying selection, one should observe decrease in the substitution rate in the longer-overlap regions compared to shorter-overlap ones – is this the case?

#### Author's response

*Dr. Gelfand wrote: "Since the frequency of candidate stop codons is not the same in the two shifted reading phases of the upstream gene, this might influence the general conclusions made in the paper." However, as shown in Figure *4, *there is no significant difference between the frequencies of stop codons in the two phases, while the frequency of start codons in phase 1 is significantly higher than that of phase 2. Regarding the stronger purifying selection in long extensions, we have clearly failed to convey the idea. All we meant was to convey the common-sense assumption that in molecular evolution "big changes" are selected against more frequently and more stringently than "small changes." The strength of the negative selection is expected to be positively correlated with the length of the extension following the obliteration of a stop codon*.

6) Background, second paragraph: "Overlaps in the same frame are rare": that depends on how one quantifies it; gene fusions do not seem to be very rare in bacterial genomes, especially conserved with long overlaps.

#### Author's response

*In our dataset, there are 187 phase-0 same-strand overlaps (0.26%). One reason for the paucity may be that in phase-0 same-strand overlaps, stop codons should be unstable or subjected to readthrough. Another reason may be the one raised by Dr. Gelfand, i.e., the ease with which gene fusion occurs in bacteria*.

7) Results, first paragraph: It might be interesting to learn more about 18 non-standard start codons yielding short phase-1 overlaps. Are they functional? Are they conserved? Are they regulatory?

#### Author's response

*True. However, these might be also a result of annotation or sequencing errors*.

8) Discussion, fifth paragraph: One of the reasons for relative scarcity of 3'-extensions might be that many bacterial genes contain tandem stop codons. This has been ascribed to avoidance of translational readthrough, but an evolutionary consequence is that mutation in one stop codon from a tandem pair does not create overlapping genes.

#### Author's response

*There is no relative scarcity of 3'-extensions. In fact, the rate of accumulation and degradation of overlapping pairs is higher for overlaps caused by mutation at the 3'-end of the upstream gene compared to overlaps caused by mutation at the 5'-end of the downstream gene *[9].

9) Discussion, sixth paragraph: Correlation between the GC-content and the frequency of stop codons in frames 1 and 2 does not prove the absence of selection for such stop codons: one needs to demonstrate that the number of observed stops coincides with the number of expected ones, while controlling for dependencies between adjacent codons.

#### Author's response

*Given that AT-rich genomes have, on average, five times more frame-shift stop codons than GC-rich genomes, we believe that the impact of selection on frame-shift stop codon frequency should be small compared to the impact of the mutation pattern that affects composition*.
